# ﻿*Lysimachia speciosa* (Primulaceae), a new species from Central China

**DOI:** 10.3897/phytokeys.263.139659

**Published:** 2025-10-02

**Authors:** Song-Zhi Xu, Han Xu, Qi-Liang Gan, Zhen-Yu Li

**Affiliations:** 1 School of Life Science, Nantong University, Nantong, Jiangsu 226019, China Nantong University Nantong China; 2 Institute of Plant Quarantine, Chinese Academy of Inspection and Quarantine, Beijing 100176, China Chinese Academy of Inspection and Quarantine Beijing China; 3 School of Pharmacy, Hubei University of Chinese Medicine, Wuhan 430072, China Hubei University of Chinese Medicine Wuhan China; 4 State Key Laboratory of Plant Diversity and Specialty Crops, Institute of Botany, Chinese Academy of Sciences, Beijing, China Chinese Academy of Sciences Beijing China

**Keywords:** China, Hubei, *
Lysimachia
*, new species, taxonomy

## Abstract

A new species, *Lysimachia speciosa*, from western Hubei Province, Central China, is described and illustrated. The species is morphologically similar to *L. deltoidea*, especially var. cinerascens, but mainly differs from the latter by the recurved pubescence on the stem, petiole, and pedicel; the rhombic-ovate or ovate-lanceolate leaf blade; the longer petiole, calyx, corolla, stamens, and style; the erose lobes of the corolla; and the hairy ovary and capsule.

## ﻿Introduction

The genus *Lysimachia* L. consists of ca. 280 species (POWO 2024), mainly distributed in the temperate and subtropical regions of the northern hemisphere, with a few species occurring in Africa, Latin America, and Oceania (Chen and Hu 1979; Chen et al. 1989; Hu and Kelso 1996). Many species in this genus have economic value, such as medicinal, ornamental, and aromatic uses. “Flora of China” recorded 138 species in China, most distributed in southwest China, especially in the Karst region (Hu and Kelso 1996; Hao and Hu 2001; Hao et al. 2004). Over the last two decades, more than 20 new species of *Lysimachia* from China have been published (Liu et al. 2014; Nong et al. 2023; Zhang et al. 2024). Central China is another region with rich *Lysimachia* flora. It consists of 44 species, of which 16 species are endemic: *L. pseudo-trichopoda* Hand.-Mazz., *L. hypericoides* Hemsl., *L. ophelioides* Hemsl., *L. nanchuanensis* C.Y.Wu ex F.H.Chen & C.M.Hu, L. phyllocephala var. polycephala (Chien) Chen & C.M.Hu, *L. fistulosa* Hand.-Mazz., *L. henryi* Hemsl., *L. liui* Chien, *L. pterantha* Hemsl., *L. crista-galli* Pamp. ex Hand.-Mazz., *L. auriculata* Hemsl., *L. crispidens* (Hance) Hemsl. (Hu and Kelso 1996), *L. xiangxiensis* D.G. Zhang, C. Mou & Y. Wu (Mou et al. 2020), *L. porcatisepala* S.R. Yi (Yi 2020), *L. brevianthera* X.W. Li (Ke et al. 2021), *L. xuyongensis* X.F. Gao & W.B. Ju (Ju et al. 2021), and *L. coriacea* S.R. Yi & H.F. Yan (Yan et al. 2022). An unknown *Lysimachia* species found and collected in Zhuxi County shows some similarities to *L. grammica* Hance. However, it has transparent glandular spots scattered on the plant instead of black glandular stripes. Additionally, the stem, petiole, and pedicel are densely recurved-pubescent rather than spreading pilose. The differences in flowers and fruits between these two species are also evident. The flowers of this unknown taxon are yellow and 5-merous, with anthers opening by lateral slits, distinguishing it from subgen. Naumburgia (Moench) Klatt (which has 6-merous or 7-merous flowers) and subgen. Idiophyton Hand.-Mazz. (which has anther opening by apical pores). Additionally, the basifixed anthers of this unknown species indicate it undoubtedly belongs to subgen. Lysimachia sect. Nummularia (Gilib.) Klatt. Upon further examination, we found that this unknown taxon is more similar to L. deltoides var. cinerascens Franch. Based on the unique combination of characteristics, we propose that this unknown species is new to science.

## ﻿Materials and methods

The voucher specimens of the putative new species were collected in Zhuxi County, Hubei Province, in 2023. Morphological observations and measurements were based on living plants in the field and specimens stored in PE, IBSC, and A (the herbaria names see Thiers 2024). Morphological comparisons with its relatives were made by consulting specimens stored in PE and some virtual specimen databases (CVH and POWO). All morphological characters were measured with dissecting microscopes and described using the terminology presented by Harris and Harris (1994). The conservation status of the new species was assessed following the IUCN guidelines (IUCN 2024).

## ﻿Results

### ﻿Taxonomic notes

The leaves of the new species are somewhat similar to those of some species from sect. Nummularia, which belongs to ser. Elatae Hand.-Mazz., ser. Phyllocephalae (Hand.-Mazz.) Chen & C.M. Hu, ser. Fordianae Chen & C.M. Hu, ser. Deltoideae Hand.-Mazz., and ser. Drymarifoliae Hand.-Mazz. However, characteristics such as plant habits, leaf arrangement, indumentum, glandular spots or stripes, inflorescence, flowers, fruits, and seeds are distinctly different between some members of these groups and the new species. For instance, the leaves of *Lysimachia xiangxiensis* D.G. Zhang, C. Mou & Y. Wu (Mou et al. 2020), which belong to ser. Elatae, are similar to those of the new species, but the former have opposite leaves and differences in habit, indumentum, flowers, fruits, and seeds. Therefore, species with both opposite and alternate leaves were selected as akin species.

### ﻿Taxonomic treatment

#### ﻿ Lysimachia speciosa

Taxon classificationPlantaeEricalesPrimulaceae

Q.L.Gan, Z.Y.Li & S.Z.Xu sp. nov.

DC0A22F4-1178-5121-8350-19106C90890C

urn:lsid:ipni.org:names:77369879-1

Fig. 1

##### Diagnosis.

*Lysimachia speciosa* is most similar to L. deltoidea var. cinerascens and *L. grammica* in having cespitose and ascending-erect stems, densely multicellular hairs on the plant, both opposite and alternate leaves, and solitary and axillary yellow flowers, but the new species can be easily distinguished from both by its recurved-pubescent indumentum on stems, petioles, and pedicels; shorter than subtending leaves pedicels; much longer calyx, corolla, filaments, and style; and pilose ovary and capsule. The diagnostic features between *Lysimachia speciosa*, L. deltoidea var. cinerascens, and *L. grammica* are summarised in Table 1.

**Table 1.** Morphological comparisons of *Lysimachia speciosa*, L. deltoidea var. cinerascens, and *L. grammica*.

Characters	*L. speciosa*	L. deltoidea var. cinerascens	*L. grammica*
Indumentum on stem, petiole and pedicel	densely recurved-pubescent	densely spreading pilose	densely spreading pilose
Glandular on stem, leaf, calyx and corolla	transparent glandular spots	transparent glandular spots	blackish or black-brown glandular stripes
Petiole	5–15 mm long	2–3 mm long or subsessile	4–15 mm long
Leaf blade	rhomboid-ovate to ovate-lanceolate, 1–4 × 0.5–2 cm	elliptic to suborbicular, 1–2.5 × 0.8–1.8 cm	ovate to rhomboid-ovate, 1.3–3.5 × 0.8–2.5 cm
Pedicel	0.5–1 cm long, shorter than subtending leaf	1–2.5 cm long, ca. as long as subtending leaf	1–4 cm long, usually shorter than subtending leaf
Calyx	9–10 mm long	4–5 mm long	ca. 7 mm long
Corolla lobes	10–11 × 6–7 mm, margin erose	4–6.5 × 4–5 mm, margin entire	5–8 × 3–5 mm, margin entire
Filaments	tube ca. 2 mm long, connate tube ca. 3 mm; free parts 4–5 mm	tube ca. 1 mm long, connate ring ca. 1 mm; free parts ca. 2 mm	tube 0.5–1 mm long, connate ring ca. 0.5 mm; free parts 1.5–2.5 mm
Anthers	oblong, ca. 2 mm long	ovate, ca. 1 mm long	oblong, ca. 2 mm long
Ovary	pilose	glabrous	pubescent
Style	ca. 6 mm long, base spreading pilose	ca. 3.5 mm long, glabrous	ca. 4.5 mm long
Capsule	ca. 3.5 mm in diam., pilose	ca. 3 mm in diam., glabrous	ca. 4 mm in diam., pubescent

##### Type.

China: • Hubei Province, Zhuxi County, Jiangjiayan Town, Yaowangmiao Village, roadside grassland, flower yellow, alt. 580 m, 20 May 2023, *Qi-Liang Gan PE23-5-2* (holotype, PE!, PE02446668; isotype, PE!, PE02446669; A, IBSC)

##### Description.

A perennial herb, 15–45 cm tall. *Rhizomes* creeping, with fibrous adventitious roots at nodes. *Stems* usually 3 to numerous, cespitose, ascending-erect, terete, with 8–20 nodes, distal internodes shorter, unbranched or shortly branched, sparsely transparent glandular spotted, densely recurved-pubescent. *Leaves* opposite on lower part, alternate on upper part; proximal and distal leaves smaller; petiole 5–15 mm long, narrowly winged, densely recurved-pubescent; leaf blade rhombic-ovate or ovate-lanceolate, 1–4 cm long, 0.6–2 cm wide, apex short-acuminate or acute, rarely obtuse, base cuneate and decurrent, margin entire, sparsely transparent glandular spotted, adaxially green, abaxially pale green, densely pubescent on both surfaces; lateral veins 2–3 on each side of midrib, alternate, inconspicuous. *Flowers* axillary in the middle and upper parts of the stem, solitary; pedicel 5–10 mm long, ascending to recurved, densely recurved-pubescent; calyx green, 9–10 mm long, 5-lobed parted almost to the base, tube ca. 0.5 mm long, lobes linear-lanceolate, 8.5–9.5 mm long, 1.5–2 mm wide in lower part, caudate at apex, sparsely transparent glandular spotted, densely pubescent outside, midrib green, convex abaxially; corolla yellow, tube ca. 2 mm long, 5-lobed, lobes broadly ovate, 10–11 mm long, 6–7 mm wide, sparsely transparent glandular spotted, margin erose; stamens 5, erect, glabrous, filaments connate basely into a tube ca. 3 mm high, free parts 4–5 mm, anthers dorsifixed, oblong, ca. 2 mm long, opening by lateral slits; ovary ovoid, densely pilose, style filiform, ca. 6 mm long, spreading pilose basely, stigma capitate, slightly wider than style. *Capsule* pale brown, subglobose, ca. 3.5 mm in diam., pilose. *Seeds* numerous, brown, ovoid in outline and irregular, 1.2–1.8 mm long, 3–4-angled, glabrous.

**Figure 1.**
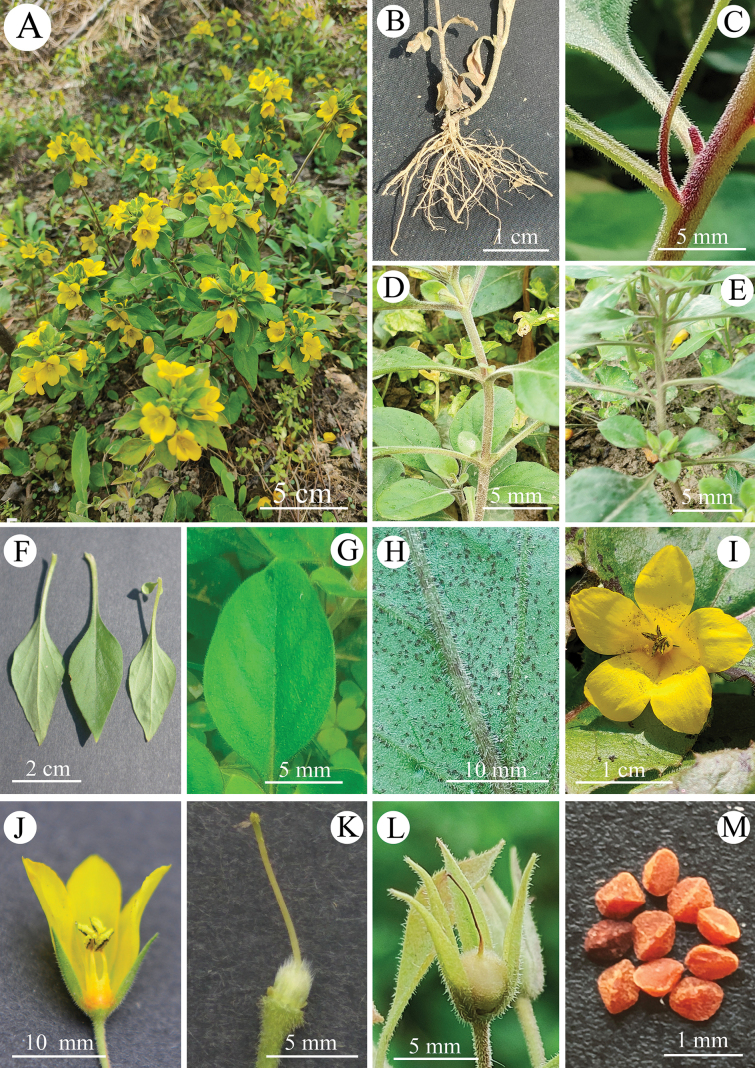
*Lysimachia speciosa* sp. nov. **A** flowering plants **B** roots **C** stem and petioles **D** opposite leaves on lower stem **E** alternate leaves on mid- and distal stem **F** leaves **G** mid-vein and lateral veins **H** abaxial surface of leaf **I** adaxial view of corolla **J** dissected corolla showing stamens **K** pistil **L** capsule and persistent calyx **M** seeds.

##### Phenology.

Flowering from May to June, fruiting from June to July.

##### Distribution and habitat.

The population of *Lysimachia speciosa* is known only from the type locality (32.322862N, 109.532557E), Yaowangmiao Village, Zhuzi County, Hubei Province, China. It grows in roadside grassland at an altitude of 580 m.

##### Etymology.

*Lysimachia speciosa* is a perennial herb with an attractive plant type and numerous cespitose, ascending-erect stems. Besides, the stem and petiole of the new species sometimes are flushed red. The flowers are yellow and showy. Thus, the specific epithet is derived from the aforementioned morphological characteristics.

##### Vernacular name.

Mei Li Guo Lu Huang (Chinese).

##### Provisional conservation assessment.

*Lysimachia speciosa* is currently known from a single population of approximately 30 individuals at its type locality in Yaowangmiao Village, Jiangjiayan Town, Zhuxi County, Hubei Province. Due to insufficient data on this species, its conservation status cannot be fully assessed at this time. Therefore, its provisional conservation status is temporarily classified as Data Deficient (DD) according to the IUCN (2024).

##### Additional specimen examined.

l.c., fruit pale brown, 14 July 2023, *Qi-Liang Gan* PE23-7-4 (PE!, PE02446670)

## Supplementary Material

XML Treatment for Lysimachia speciosa

## ﻿Additional information

### Author ORCIDs

Song-Zhi Xu https://orcid.org/0000-0002-4178-5559

### Data availability

All of the data that support the findings of this study are available in the main text.
